# Anti-spasmodic action of crude methanolic extract and a new compound isolated from the aerial parts of *Myrsine africana*

**DOI:** 10.1186/1472-6882-11-55

**Published:** 2011-07-06

**Authors:** Sadiq Azam, Shumaila Bashir, Bashir Ahmad

**Affiliations:** 1 Pharmabiotech Research Lab, Centre for Biotechnology and Microbiology, University of Peshawar, Peshawar, Khyber Pakhtunkhwa, Pakistan; 2 Department of Pharmacy, University of Peshawar, Peshawar, Khyber Pakhtunkhwa, Pakistan

**Keywords:** *Myrsine africana*, Antispasmodic action, Myrsigenin, Rabbit jejunum, KCl induced contractions

## Abstract

**Background:**

*Myrsine africana *is an herbaceous plant that is traditionally used as appetizer and carminative. Locally, it is used for the treatment of pulmonary tuberculosis, rheumatism and diarrhea by healers. The aims of the current study were to screen the crude methanol extract obtained from the aerial parts (leaves and stem) of *M. africana*, for antispasmodic actions on isolated tissues and further to subject the ethyl acetate (EtOAc) fraction of plant to column chromatography for isolation of pure compounds.

**Methods:**

The antispasmodic action of the crude methanol extract was measured on the spontaneous rabbit's jejunum preparations at concentration 0.01, 0.03, 0.1, 0.3, 1.0, 5.0 and 10.0 mg/ml. The crude extract was also applied, in similar concentrations, on KCl (80 mM) induced contractions to explain its possible mode of action.

**Results:**

A new compound Myrsigenin was isolated from the EtOAc fraction of *M. africana*. The structure of the compound was identified with the help of ^13^C-NMR, ^1^H-NMR, HMBC, HMQC, NOESY and COSY. The plant crude methanol extract showed a significant antispasmodic action on rabbit jejunum and abolished the tissue contraction completely at concentration of 5.0 mg/ml.

**Conclusion:**

The study concludes that the methanol crude extract of aerial parts of *M. africana *has antispasmodic action possibly through the calcium channel blocking mechanisms. A new compound Myrsigenin was isolated from the EtOAc fraction of the plant.

## Background

Plants play an important role in many cultures. Human being uses these plants in the form of sheltering, clothing, feeding, hunting and nursing for their basic requirements. Plants are the basic source of medicines in the traditional system that provides mankind new remedies for different disease(s). Recently interest in the folk medicine has highly increased and gained a scientific basis for the appropriate use in official medicines [1]. Due to unique biodiversity and salubrious climate, Pakistan is quite rich in medicinal herbs which are scattered over a large area. There are up to 6,000 wild plant species out of which 400-600 are medicinally important [2]. In continuation to our previous work [3-5] the present work was carried out on *M. africana *(Myrsinaceae), locally called Babrang. Myrsinaceae is a large family of about 35 genera and 1000 species, widely distributed in tropical and subtropical regions [6]. Traditionally *M. africana *is used as fragrance in tea, appetizer, carminative, spices and flavoring agent. The fruits are edible and locally used as an anthelmintic. It is also used for the treatment of rheumatism, pulmonary tuberculosis, diarrhea, hemorrhage and toothache [7-11]. In powder form, the dried fruits of the plants are taken daily (once at bedtime) with a cup of curd for 3-4 days for intestinal worms. Decoction of fresh leaves is taken orally daily (twice) for 6-7 days during scanty urination, skin allergy, boils and to purify blood [12]. It has been reported that the chloroform (CHCl_3_) fraction of *M. africana *possess moderate phytotoxic activity (31.25%) against *Lemna minor *L at higher concentration [4]. The methanol extract and CHCl_3 _fraction showed good antibacterial activity against *Klebsiella pneumoniae *(MIC_50 _= 2.45 and 2.1 mg/ml, respectively). A moderate haemagglutination effect was shown by the methanol extract and CHCl_3 _fraction against human red blood cells (RBCs) of blood group AB^- ^and same moderate effect was observed for aqueous fraction against AB^+ ^[4]. Significant inhibitory activities were shown by the alcoholic extract of leaves and twigs of the plants against walker intramuscular carcinosarcoma in rats [13]. The mixture of leaves and dried fruits of the plant in water has shown 77% efficacy against Trichostrogylus, Haemonchus and Oesophagostomum spps [14].

The phytochemical investigations revealed that the plant contains various groups of natural compounds; triterpenes, benzoquinones, flavonoids and steroids [15-20]. This manuscript deals with isolation and characterization of the compound with a six membered rings E and five membered F ring spiro fused compound isolated from *M. africana *and screening of the crude methanol extract of plant for anti-spasmodic action on isolated rabbit's jejunum.

## Methods

### General Experimental Procedure

Jeol JMS 600 and HX 110 mass spectrometers with the data system DA 5000 were used to record HREI-MS. The ^1^H-NMR spectrum was recorded in CDCl_3 _on Bruker AMX-400 and AMX-500 NMR spectrometers with TMS as an internal standard using a UNIX operating system at 400 and 500 MHz, respectively. The ^13^C-NMR spectrum was recorded at 100 MHz on a Bruker AMX-500 NMR spectrometer in CDCl_3_. The [α]D was recorded on Jasco P-2000 polarimeter. JASCO J-810 spectropolarimeter was used to record CD spectrum. Silica gel (230-270 mesh) was used for Column Chromatography (CC). Silica gel coated TLC card (GF-254, 20 × 20 cm, 0.25 mm thick, Merck) were used to check the purity of compound and were observed under UV light (254 and 366 nm) and ceric sulphate was used as a spraying reagent.

### Collection, Extraction and Fractionation

The whole aerial parts (leaves and stem) of *M. africana *were collected from Attar Sheesha, Hazara division, in December 2007-January 2008. Prof. Dr. Habib Ahmad, Plant Taxonomist, Hazara University, Khyber Pakhtunkhwa, identified the plant.

The plant materials were shade dried, chopped and ground to fine powder by using electric grinder. The powdered materials (7.6 kg) were then soaked (twice) in commercial grade methanol for 15 days at room temperature with occasional shaking. After 15 days methanol soluble materials were filtered off. All the filtrates were concentrated under vacuum at 40°C using a rotary evaporator till a blackish crude methanol extract was obtained.

The crude methanol extract obtained was suspended in distilled water (400 ml) and partitioned with *n*-hexane (3 × 400 ml), CHCl_3 _(3 × 400 ml), EtOAc (3 × 400 ml) and BuOH (3 × 400 ml) to yield *n*-hexane (50 g), CHCl_3 _(45 g), EtOAc (255 g), BuOH (190 g) and aqueous (210 g) fractions. 50 gram of crude extract was reserved for pharmacological/biological activities.

### Effects On Rabbit's Jejunum Preparations

Our present study was done on isolated rabbit's jejunum tissues to explore the plant extract for possible spasmolytic activity.

### Drugs, standards and solution preparation

Acetylcholine (BDH Chemicals, Poole, England), Atropine and rest of the chemicals (E. Merck Germany) used in the experiments were of analytical grade. All solutions were freshly prepared in distilled water on the same day of the experiment.

Stock solutions of acetylcholine (10^-2^μM) and KCl were prepared. The acetylcholine solution was diluted upto 10^-4^μM by serial dilution of the stock solution and final concentration of the KCl solution was made 80 mM in bath solution. The stock solution of sample (crude methanol extract) was prepared in distilled water by suspending 300 mg/ml and serially diluted up to 30 and 3 mg/ml.

### Animals and data recording

Rabbits either male or female (local breed; weight in Kg 1.0 - 1.4) were used in the experiments. The rabbits were maintained at the "Animal House of University of Malakand" according to the standards mentioned in the "Animals Bylaws 2008 of the University of Malakand (Scientific Procedures Issue-1)". The ethics committee of the University of Malakand approved this study (Vid case # UOM/EC/02/2008). The protocols comply with the Byelaws 2008 of the University of Malakand that deal with scientific procedures for experimental work on animals. All of the rabbits were fed with standard diet and tap water. They were given only water, 24 hours prior to start experiments. The tissue responses were noted by Teaching Force Transducer (Model No: MLT 0210/A Pan Lab S.I.) attached with Power lab (Model No: 4/25 T) ADInstruments, Australia. Range for recording the data was 20 mv, Low pass 5 Hz × 10 gain using input 1 at rate 40/S.

### Rabbit's jejunum preparations

The tissue preparation for the experiments was carried out through following procedure [21].

1. The rabbits maintained at animal house were sacrificed and the jejunum portion(s) of the rabbits were isolated. The carbogen gas (5% CO_2 _and O_2 _mixture) was used to aerate these isolated tissues placed in Tyrode's solution. The constituents and their concentrations (mM) used in Tyrode's solution were: KCl 2.68, NaCl 136.9, MgCl_2 _1.05, NaHCO_3 _11.90, NaH_2_PO_4 _0.42, CaCl_2 _1.8 and glucose 5.55 [22-25].

2. 1.5 cm pieces of jejunum, maintained in the Tyrode's, were mounted in 10 ml tissue bath maintained at 37°C in Tyrode's solution aerated with carbogen gas.

3. Tension (one gram) was applied on tissue. Earlier, by giving the sub-maximal doses of acetylcholine (0.3 μM) to the tissue for keeping it undisturbed and stabilized for an equilibrium period of 30 minutes and to produce a reproducible response (normal response).

4. The plant crude methanol extract at concentration(s) of 0.01, 0.03, 0.1, 0.3, 1.0, 5.0 and 10 mg/ml were tested for the possible spasmolytic activity throughout experiments.

### Spasmolytic activity

The highly concentrated K^+ ^solution (80 Mm) was used to treat tissues in order to depolarize it and to get them in position of sustained concentration [26]. The test samples were then applied on those pre-treated tissues mounted in tissue bath, in cumulative manner, to obtain a concentration dependent response curve and their relaxation was expressed as percent of the K^+ ^induced contractions [27].

## Results and Discussion

A new compound Myrsigenin was isolated as white powder from the EtOAc fraction of *M. africana *eluting with EtOAc/*n*-hexane (0.3:9.7) as mobile phase on flash silica column. Its optical rotation, {[α]_D_^25 ^= -16.10 (MeOH, c = 0.2)} indicated the presence of chiral center in molecule. HR-ESIMS displayed quasi molecular ion at m/z 415.3217, corresponding to the formula C_27_H_42_O_3 _(cal. C_27_H_42_O_3 _+ H = 415.3212).

^1^H-NMR spectrum exhibited resonances at δ 0.72 d (*J*_21_, _20 _= 6.5 Hz), 0.80 (singlet), 0.95 (d, *J*_27_,_24 _= 6.5 Hz) and 1.06 ppm (singlet), which were assigned to the C-21, C-19, C-27 and C-18 methyl protons, respectively. Downfield methine protons at δ 3.44 (m, broad) (W_1/2 _= 12.5 Hz) and 4.39 (q, J = 6.5 Hz) were attributed to the H-3 and H-16 respectively. Resonances at δ 3.35 (m) and 3.42 (m) were ascribed to the methylene protons of the C-27: this revealed the presence of oxygenated methylene carbon in molecule. A down field signal resonated at δ 5.33 br d (*J *_6, 7 _= 5.0), was due to the H-6.

**Figure 1:**
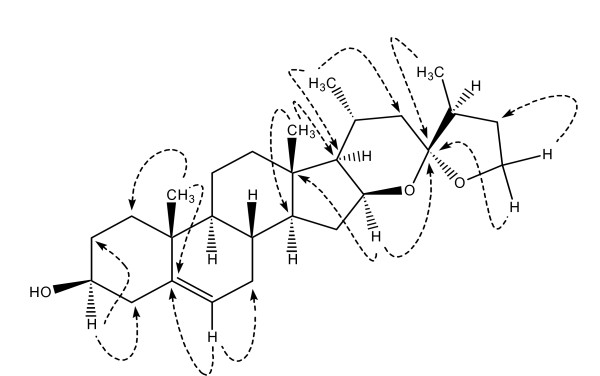
**Key HMBC interactions in Myrsigenin**.

^13^C-NMR spectrum (BB, DEPT) displayed twenty seven carbons including: four methyl, ten methylene, nine methine and four quaternary carbon atoms (Table 1). ^1^H and ^13^C-NMR clearly indicated that the compound has steroidal skeleton. ^13^C-NMR values at δ 67.9 (CH_2_), 82.2 (CH) and 110.5 (q C), indicated the presence of dioxygenated spiro ring as a side chain of the molecule. Structure of compound was further confirmed with the help of two dimensional NMR spectroscopic techniques including: COSY, HSQC, HMBC and NOESY. COSY and HMBC revealed that ring E is a six membered, while the ring F is a five membered. H-16 showed cross peaks in COSY spectrum with H-17, which in turn showed correlation with H-20, this spin system extended up to the C-22 methylene protons. This clearly indicated that ring E is six membered. Similarly, H2-26 showed cross peaks on COSY spectrum with H_2_-25, which in turn showed correlation with H-24, this spin system extended up to C-27 methyl protons, this further supported that ring E is six membered and ring F is five membered. Different spin systems, hetero atoms and functionalities were assembled using HMBC interactions. H-16, H_2_-26 and H_3_-27 displayed HMBC correlation with C-23. While H_3_-21 exhibited correlation with C-22, C-20 and C-17. Key HMBC interactions in compound are shown in Figure 1.

**Table 1:** ^1^H and ^13^C-NMR and Chemical Shifts of compound (ppm, CDCl_3_, 500 and 100 MHz, respectively).

C. No	Multiplicity (DEPT)	^13^C-NMR	^1^H (*J *= Hz)
1	CH2	38.5	1.71(m # of H), 1.93(m, #of H)
2	CH2	32.3	1.59 m, 1.83 m
3	CH	72.4	3.44 br m (*w*_1/2 _= 12.5)
4	CH2	43.0	2.21 dd, 2.23 dd
5	C	140.8	------
6	CH	121.8	5.53 br d (5.0)
7	CH2	32.8	1.81 m, 1.97 m
8	CH	31.8	1.61 m
9	CH	51.7	0.95 m
10	C	36.5	------
11	CH2	22.0	1.01 m, 1.42 m
12	CH2	40.9	1.69 m, 1.40 m
13	C	41.3	------
14	CH	57.8	1.09 m
15	CH2	32.7	1.49 m, 1.63 m
16	CH	82.2	4.39 q like (7.5)
17	CH	63.7	1.75 m
18	CH3	19.8	1.06 s
19	CH3	14.9	0.80 s
20	CH	29.9	1.60 m
21	CH3	17.5	0.72 d (*J*_21,20 _= 6.5)
22	CH2	33.2	1.2 dd (overlapped), 1.50 dd (overlapped)
23	C	110.5	------
24	CH	42.9	1.84 dd (5.5, 4.5)
25	CH2	32.4	1.65 m, 1.81 m
26	CH2	67.9	3.35 m, 3.42 m
27	CH3	16.8	0.95 d (*J*_27,24 _= 6.5)

**Figure 2:**
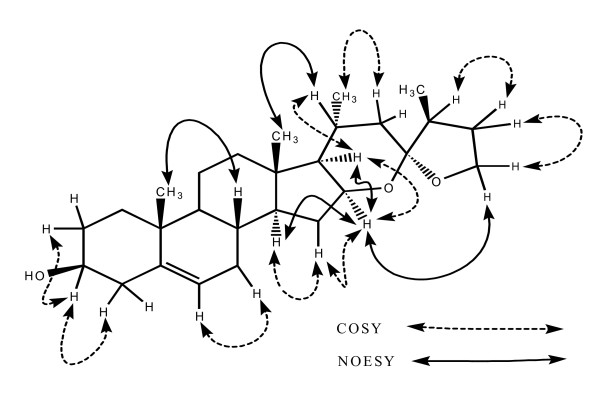
**Key NOESY and COSY interactions in Myrsigenin**.

Stereochemistry in compound was assigned on the basis of biogenesis pathway, coupling constant values and NOESY spectroscopy. H-16 displayed NOESY interactions with H-26, H-14 and H-17, this indicated that *cis *ring fusion between ring D and E, and H-16 and H-26 are close in space. Key NOESY interactions in compound are shown in Figure 2. CD spectrum indicates an intense positive cotton effect at 210 nm. This type of six membered rings E and five membered F ring spiro fused compound is not so common, this compound is believed to have been biosynthesized from C-27 [28].

### Effects On Rabbit'S Jejunum Preparations

#### Spasmolytic activity

**Figure 3:**
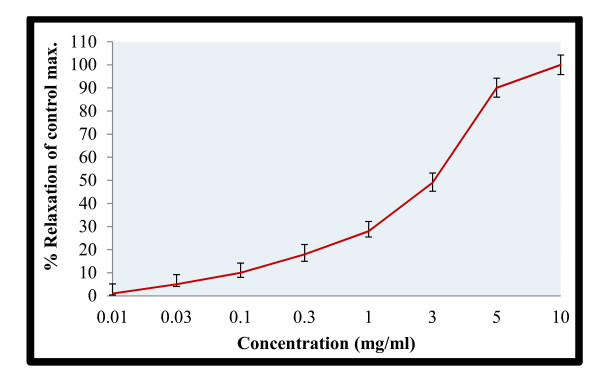
**Spasmolytic activity of *M. africana *on spontaneous rabbit's jejunum preparations**.

It is evident from Figure 3 that methanol extract produced a concentration dependent fall in spontaneous rabbit's jejunum preparations. The extract totally abolished the spontaneous contractions of tissues at concentration 5.0 mg/ml. This confirms that the plant specie contains spasmolytic constituents and therefore has antispasmodic action.

**Figure 4:**
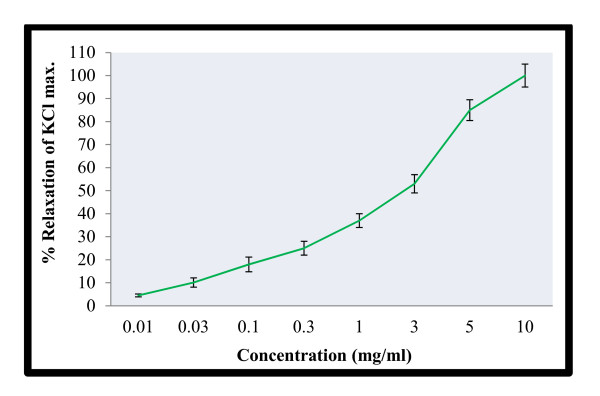
**Spasmolytic activity of *M. africana *on KCl (80 mM) induced contractions on rabbit's jejunum preparations**.

**Figure 5:**
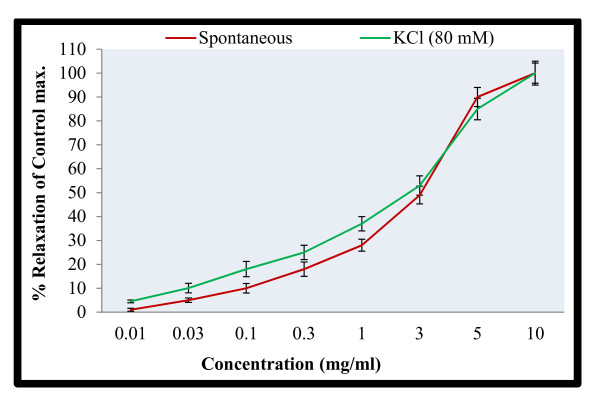
**Spasmolytic activity of *M. africana *on spontaneous and KCl (80 mM) induced contractions on rabbit's jejunum preparations**.

In order to explain the possible mode of actions, we tried the extract on contractions produced by high potassium concentration (80.0 mM). The results are shown in Figure 4. Interestingly, the extract produced similar type of actions in the KCl-induced contractions. The results of the higher concentration are superimposable (Figure 5). Thus the extract is having almost similar values of EC_50 _for the spontaneous contractions and KCl-induced contractions. Studies have shown that high KCl induced contractions are due to membrane depolarization of the smooth muscles of intestine. Depolarization of the muscles by KCl (80 mM) opens the voltage operated calcium channels and allows the extra-cellular calcium into the cytosol [22,24]. This is followed by the release of calcium from internal stores that help in sustained contractions. In other word KCl induced contractions are mediated by both extracellular and intracellular calcium levels [29]. The results suggest that the plant specie is having antispasmodic action possibly through the calcium channel blocking mechanisms [30].

## Conclusion

The present study reveals the antispasmodic action of crude methanol extract of *M. africana *and isolation of a new steroidal compound "Myrsigenin" from this specie.

## Competing interests

The authors declare that they have no competing interests.

## Authors' contributions

SA (PhD research scholar) carried out sampling and experimental work, BA supervised the research work conducted by SA and designed the experimental work and manuscript preparation with the help of SB and SA. The final manuscript is approved by all of the authors after reviewing it critically.

## Pre-publication history

The pre-publication history for this paper can be accessed here:

http://www.biomedcentral.com/1472-6882/11/55/prepub
